# Western diet enhances benzo(a)pyrene-induced colon tumorigenesis in a polyposis in rat coli (PIRC) rat model of colon cancer

**DOI:** 10.18632/oncotarget.7901

**Published:** 2016-03-03

**Authors:** Kelly L. Harris, Stephanie R. Pulliam, Emmanuel Okoro, Zhongmao Guo, Mary K. Washington, Samuel E. Adunyah, James M. Amos-Landgraf, Aramandla Ramesh

**Affiliations:** ^1^ Department of Biochemistry and Cancer Biology, Meharry Medical College, Nashville, TN 37208, USA; ^2^ Department of Physiology, Meharry Medical College, Nashville, TN 37208, USA; ^3^ Department of Pathology, Vanderbilt University, Nashville, TN 37232, USA; ^4^ Department of Veterinary Pathobiology, University of Missouri, Columbia, MO 65211, USA

**Keywords:** benzo(a)pyrene, colon cancer, PIRC rat, western diet, polyp

## Abstract

Consumption of Western diet (WD), contaminated with environmental toxicants, has been implicated as one of the risk factors for sporadic colon cancer. Our earlier studies using a mouse model revealed that compared to unsaturated dietary fat, the saturated dietary fat exacerbated the development of colon tumors caused by B(a)P. The objective of this study was to study how WD potentiates B(a)P-induced colon carcinogenesis in the adult male rats that carry a mutation in the *Apc* locus - the polyposis in the rat colon (PIRC) rats. Groups of PIRC rats were fed with AIN-76A standard diet (RD) or Western diet (WD) and received 25, 50, or 100 μg B(a)P/kg body weight (wt) via oral gavage for 60 days. Subsequent to exposure, rats were euthanized; colons were retrieved and preserved in 10% formalin for counting the polyp numbers, measuring the polyp size, and histological analyses. Blood samples were collected and concentrations of cholesterol, triglycerides, glucose, insulin and leptin were measured. Rats that received WD + B(a)P showed increased levels of cholesterol, triglycerides, and leptin in comparison to RD + B(a)P groups or controls. The colon tumor numbers showed a B(a)P dose-response relationship. Adenomas with high grade dysplasia were prominent in B(a)P + WD rats compared to B(a)P + RD rats and controls (p < 0.05). The larger rat model system used in this study allows for studying more advanced tumor phenotypes over a longer duration and delineating the role of diet - toxicant interactions in sporadic colon tumor development.

## INTRODUCTION

Colorectal cancer (CRC) is one of the most common cancers in the Western world. In the United States alone, nearly 136,830 new cases of CRC have been diagnosed, and 50,310 deaths will occur due to this cancer this year alone [1]. Epidemiological studies have shown that environmental factors, and especially diet, play an important role in susceptibility to sporadic colon cancer [2]. These studies estimate that diet contributes to up to 80% of documented colorectal cancer (CRC) cases. One major contributor to sporadic CRC are toxicants that are consumed through food, especially those rich in saturated fats. Heterocyclic amines (HCAs) and polycyclic aromatic hydrocarbons (PAHs) are such known food contaminants and are major causative agents of colon, lung, breast, and prostate cancers [3, 4]. With at least 20 PAHs detectable in most dietary items [5], it is no surprise that they have been reported to have an impact on obesity due to their highly lipophilic nature and presence in foods that are high in saturated fats [6]. This necessitates using a prototypical PAH compound such as benzo(a)pyrene [B(a)P] to understand the mechanisms involved in development of colorectal cancer by PAHs. In exposed individuals, B(a)P is metabolized by cytochrome P450 into metabolites, many of which are highly reactive and can form to DNA-adducts [7], predisposing the individual to cancer.

Specific components of Western diet including consumption of red meat and saturated dietary fat, and excessive adiposity have been proposed to increase susceptibility to colorectal neoplasia [8, 9]. Our group [10] has previously shown that dietary fat, especially saturated fat, enhances the development of colon tumors caused by B(a)P. Our studies [10] documented an increase in colon adenomas containing high-grade dysplasia in mice fed saturated fat. Additionally, our studies [10] confirm that B(a)P is an ideal compound to investigate the mechanism of toxicant-induced carcinogenesis in colon as this chemical is the most extensively characterized PAH compound, and studied in terms of its toxicity. Biotransformation of B(a)P produce reactive oxygen species in cells, leading to production of inflammatory cytokines [11], and genotoxic DNA damage [12].

Colon cancers arise from inherited syndromes (familial risk) and sporadic (diet, environmental toxicants etc.) factors. Of the familial forms of CRC, familial adenomatous polyposis (FAP; associated with germ line mutations in genes such as APC) and hereditary non-polyposis colon cancer (HNPCC; associated with DNA mismatch repair enzymes) are the most common. In 90% of the colon cancer cases, there is no familial history of colon cancer, however, sporadic colorectal cancer has many of the same genetic mutations, especially in the APC gene, that are somatically acquired during the life of an individual. The majority of sporadic and FAP colonic tumors involve mutations that inactivate the *APC* gene [13]. Mouse strains carrying mutagen-induced or targeted mutations in the ortholog *Apc* develop intestinal adenomas [14]. The majority of the transgenic colon cancer-specific mouse strains have a short lifespan and do not possess enough tissue mass to enable longitudinal studies on the interaction of diet and toxicants on colon carcinogenesis. To overcome these limitations, we have chosen the polyposis in rat colon (PIRC) rat model for our studies. PIRC rats develop the majority of their tumors in the colon, live 7-12 months, and can show progression to adenocarcinoma, providing a platform to study long-term effects of toxicants and administration of B(a)P and diets with varying fat content [15].

Our objective was to validate how Western diet in conjunction with B(a)P exposure affects parameters of toxicological relevance and evaluate tumor (polyp) numbers, size, and histological features in the colon of PIRC rats receiving B(a)P and Western diet in comparison to the control group fed a normal diet. Additionally, it provides insight into various biochemical and inflammatory factors that favor colon tumor development in the PIRC rat model.

## RESULTS

### Benzo(a)pyrene treatment-related effects on body weight and food consumption

**Figure 1:**
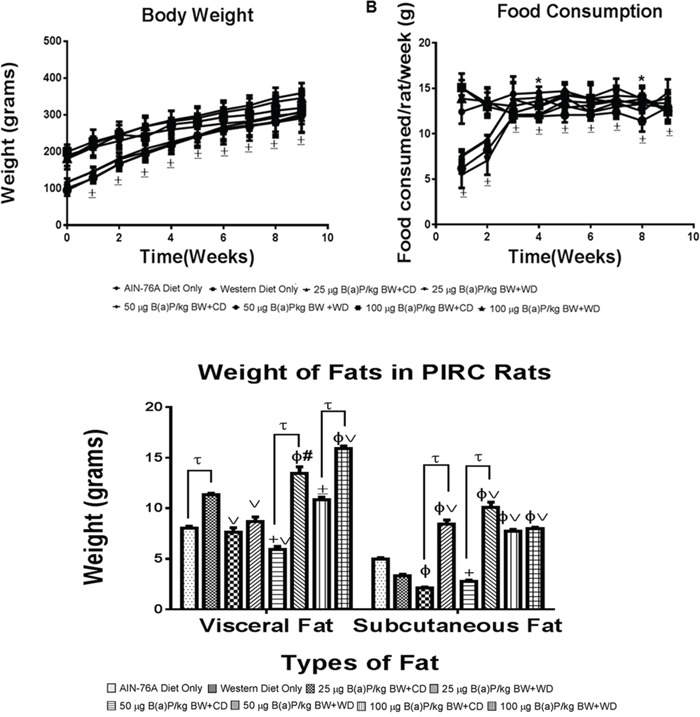
**A. Mean weekly body weights and B. food consumption of PIRC rats from AIN-76 diet and Western diet groups.** Rats were weighed prior to initiating the study and weekly thereafter. Food consumption by each group were measured over the 9 weeks. **C.** Weight of visceral and subcutaneous fats from PIRC rats following treatment with 25, 50, 100 μg B(a)P/kg body weight for 60 days via oral gavage and consumption of AIN-76A or Western diet. Annotations denote statistical significance (*p<0.05, ^±^p<0.001,^t^p<0.0001, ^+^p<0.01 compared to AIN-76A Diet Only, ^Φ^p<0.0001 compared to AIN-76A Diet Only, ^#^p<0.01 compared to Western Diet Only, and ^v^p<0.001 compared to Western Diet Only) among diets and B(a)P concentrations used.

There were no significant treatment-related changes seen in either body weight or food consumption (Figure 1A & 1B) of rats administered with B(a)P compared to controls. Although some changes in body weight and food consumption were noticed, these changes were observed within each treatment group over the 60 day exposure. These results prompted us to investigate if diet type and B(a)P dose had an influence on adipose tissue mass accrued in the body of the PIRC rats. As demonstrated in Figure 1C, PIRC rats that received B(a)P treatment along with the WD had increased adipose depots of both visceral and subcutaneous fats compared to their counterparts that received RD. Rats that were administered the 100 μg B(a)P/kg bw + WD showed the highest increase of visceral fat depots but for subcutaneous fat, the 50 μg B(a)P/kg bw + WD dose group showed the highest increase.

### Benzo(a)pyrene treatment-related effects on polyp size

**Figure 2:**
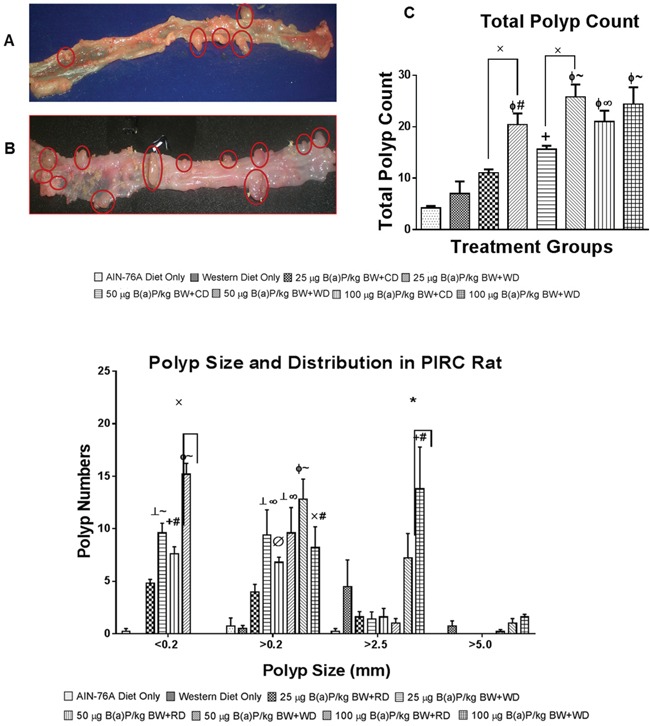
**Representative pictures of PIRC rat colons treated with A. 50 μg B(a)P/kg body weight and AIN-76A diet and B. 50 μg B(a)P/kg body weight and Western diet for 60 days via oral gavage in tricaprylin. C.** Total number of polyps in the colon of PIRC rat following treatment with 25, 50, and 100 μg B(a)P/kg bw for 60 days via oral gavage and consumption of AIN-76A diet or Western diet. **D.** Distribution and size of polyps in the colon of PIRC rat following treatment with 25, 50, and 100 μg B(a)P/kg BW for 60 days via oral gavage and consumption of AIN-76 diet or Western diet. Annotations denote statistical significance (*p<0.05, ^x^p<0.01,^+^p<0.01 compared to AIN-76A Diet Only, ^┴^p<0.001 compared to AIN-76A Diet Only, ^Φ^p<0.0001 compared to AIN-76A Diet Only, ^Ø^p<0.05 compared to Western Diet Only, ^#^p<0.01 compared to Western Diet Only, ^∞^p<0.001 compared to Western Diet Only, and ^~^p<0.0001 compared to Western Diet Only) among diets and B(a)P concentrations used.

The size and number of adenomas in colon were recorded. Representative pictures of polyps in both diets + B(a)P exposure are shown in Figure 2A. Although polyp formation was observed in rats of each group, number of polyps was higher in rats, which consumed the WD and in comparison to those receiving the RD (Figure 2B & 2C). The highest number of polyps was seen in rats receiving WD and treated with 50 μg B(a)P/kg bw. The majority of polyps that were formed among the treatment groups measured less than <.2mm, however, polyps greater than 2.5mm were seen among each group. There was an inverse correlation of increased size of polyps with decreased polyp numbers, however, in rats that received 100 μg B(a)P/kg bw and the WD, polyp sizes greater than 5mm in size were recorded with a larger than average tumor count (Figure 2D).

### Benzo(a)pyrene treatment-related effects on polyp tissue pathology & immunohistochemistry

**Figure 3:**
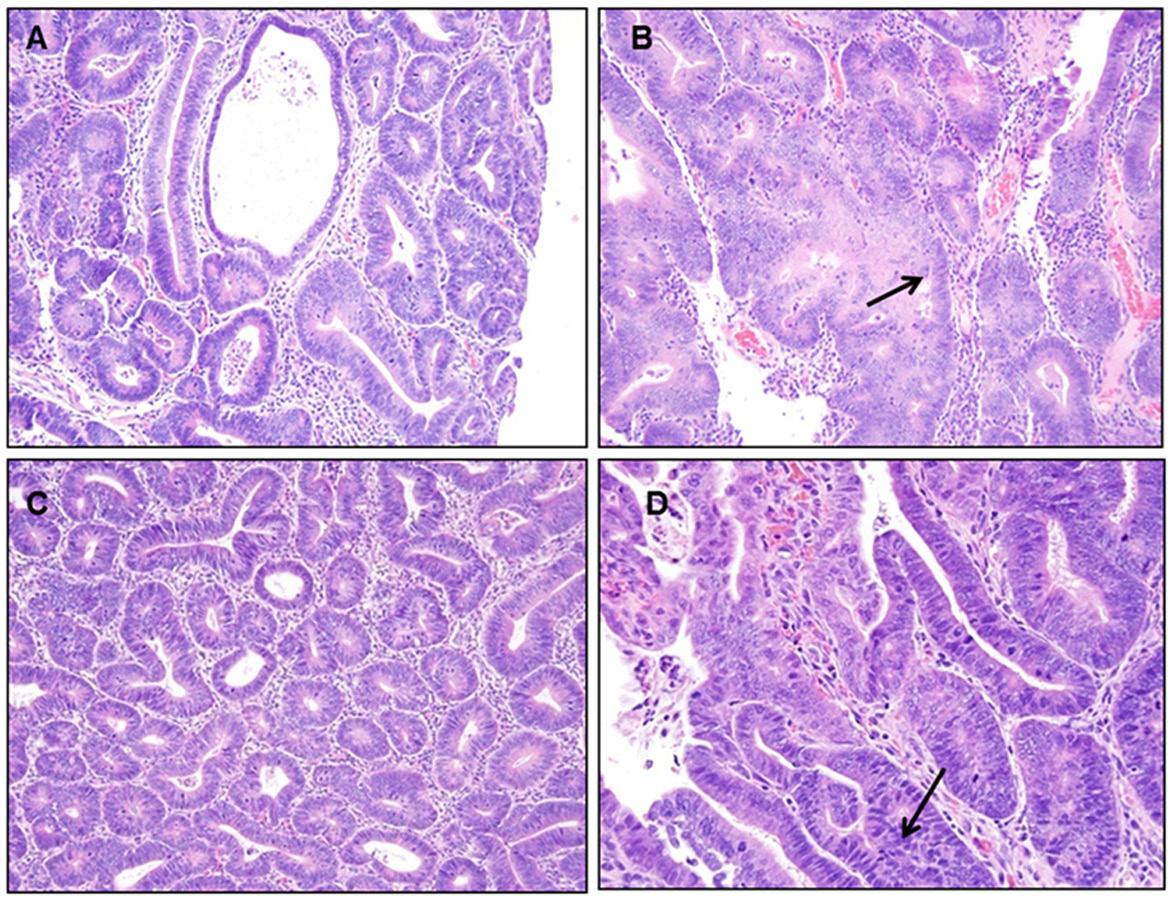
**Representative figures of H&E staining of colon of PIRC rats (200x magnification) following: A.** treatment with 25 μg B(a)P/kg body weight+AIN-76 diet **B.** treatment with 50 μg B(a)P/kg body weight+ AIN-76 diet **C.** treatment with 25 μg B(a)P/kg body weight + Western diet and **D.** treatment with 50μg B(a)P/kg body weight + Western diet. Low grade dysplasia was seen in **A** & **C.** High grade dysplasia was seen in **B** & **D.**

Representative pathological features of the colons from 2 of the 4 groups of rats treated with 25 and 50 μg B(a)P/kg bw and each diet are shown in Figure 3. Although adenomas in rats treated with B(a)P (25 μg/kg and 50 μg/kg) and fed an AIN-76A diet (Figure 3A and 3B), showed only low grade dysplasia without progression to high grade dysplasia or carcinoma, rats treated with B(a)P (25-, 50- and 100 μg/kg bw) in combination with the WD showed adenomas with high grade dysplasia. Rats that were given 50 μg/kg of B(a)P in conjunction with WD (Figure 3D), developed multiple adenomas in the colon that were of various sizes (small to large) with low to high grade dysplasia. The only exception were polyps greater than 5mm in size that were seen in the 100 μg/kg dose group. Analysis of rats treated with 25 μg/kg B(a)P + RD showed formation of multiple microscopic adenomas as well as grossly visible adenomas with low grade dysplasia (Figure 3C). Rats that were treated with 50 μg/kg B(a)P and provided with the WD demonstrated to have multiple adenomas with high grade dysplasia (Figure 3D). Similar trends of multiple adenomas with high grade dysplasia were seen in PIRC rats administered 100μg/kg B(a)P + WD (data not shown). Rats that were provided diets without B(a)P treatment did not show significant differences in colon pathology (data not shown). Histopathological evaluation of the liver tissues revealed that hepatic fat change (steatosis) was slight to moderate in rats fed either RD or WD regardless of the B(a)P dose administered. Control rats also showed a similar trend.

**Figure 4:**
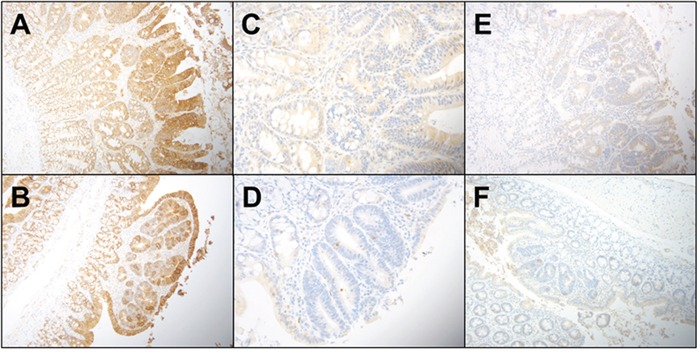
**Representative immunohistochemistry profiles of colon tissues from PIRC rats treated with diet controls: A.** E cadherin AIN-76A diet only **B.** E cadherin Western diet only adenoma **C.** Caspase 3 AIN-76A diet only **D.** Caspase 3 Western diet only **E.** TGF-β AIN-76A diet only and **F.** TGF-β Western diet only.

**Figure 5:**
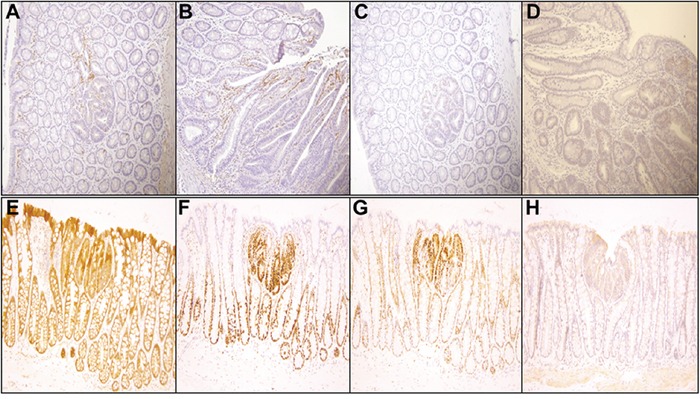
**Representative immunohistochemistry profiles of colon tissues from PIRC rats treated with 100 μg B(a)P/kg body weight + Western diet (200x magnification): A.** Cox-2 small adenoma **B.** Cox-2 large adenoma **C.** C-myc small adenoma **D.** C-myc large adenoma **E.** β-catenin **F.** PCNA **G.** cyclin D1 and **H.** TGF-β.

In order to ascertain that B(a)P-induced colon cancer is exacerbated by diet, we have investigated the localization of key signaling and inflammatory molecules in colon adenomas and non-tumor areas by immunohistochemistry. Representative figures from immunohistochemistry analysis of colons harvested from PIRC rats were shown in Figures 4 & 5. Analysis of cyclin D1, TGF-β, and beta catenin expression shows that cyclin D1 expression was increased in all adenomas, regardless of diet. TGF-β showed a slight increase in cytoplasmic positivity in adenomas, compared to normal mucosa, in all groups. Most adenomas, even very small ones, showed accumulation of beta catenin (β) in nuclei. Virtually all adenoma cells were positive for the cell proliferation marker PCNA. There was a consistent pattern of strong Cox-2 expression in stromal cells in superficial areas of adenomas, in mononuclear cells and spindled cells representing myofibroblasts in larger polyps. Weaker Cox-2 expression was noted in normal mucosa, in mononuclear cells.

**Figure 6:**
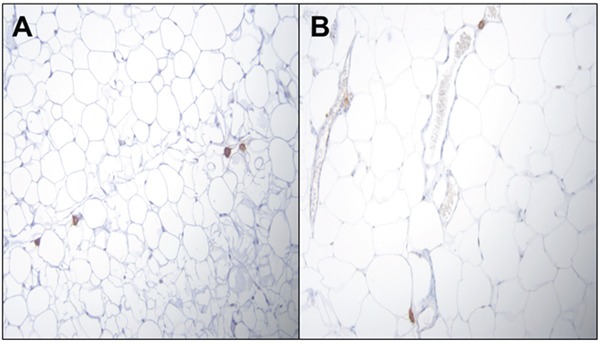
**Representative figures of H&E staining of PIRC rat adipose tissue (200x magnification) following: A.** treatment with 50μg B(a)P/kg body weight+AIN-76 diet **B.** treatment with 50 μg B(a)P/kg body weight + Western diet. There is no difference between macrophage numbers in visceral fat (VP) depots of rats.

Representative features of adipose tissue from PIRC rats fed with AIN-76A diet or Western diet were shown in Figure 6. In order to determine whether adipose depots harbor macrophage population, we counted the macrophages. While there is no difference between macrophage numbers between retroperitoneal (RP) and visceral fat (VP) depots of rats fed RD at 50 μg/kg dose group (29 versus 27 macrophages per 20 20X fields in RP and VF respectively), there is marked difference in rats fed WD at the same B(a)P dose group (15 versus 28 macrophages per 20 20X fields in RP and VF respectively).

### Benzo(a)pyrene treatment-related effects on biomarkers

Since liver plays a critical role in detoxification, we were interested in finding out whether B(a)P exposure compromised liver function. Hence we determined the alanine aminotransferase (ALT) and aspartate aminotransferase (AST) activities as markers of liver injury. The ALT and AST activities in rats from the B(a)P-treatment groups showed a slight increase over the control groups. However, the enzyme activities were comparable between B(a)P-treated rats that were fed RD and WD but none of the data were statistically significant (data not shown).

**Figure 7:**
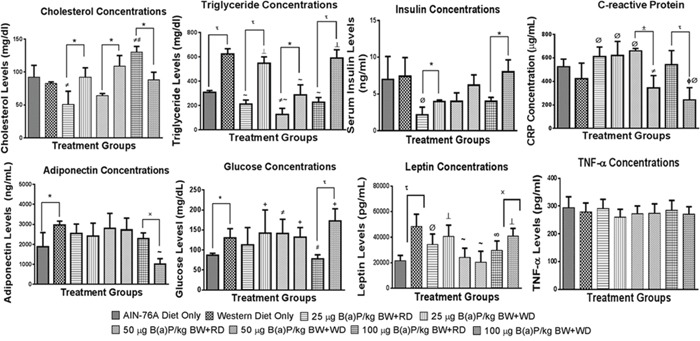
Concentrations of some biochemical markers in plasma from PIRC rats following treatment with 25, 50, and 100 μg B(a)P/kg body weight for 60 days via oral gavage and consumption of AIN-76A or Western diet Annotations denote statistical significance (*p < 0.05, *p < 0.01, ±p < 0.001, *p < 0.0001, ^#^p < 0.05 compared to AIN-76A diet only, ^ϕ^p < 0.0001 compared to AIN-76A diet only, ^Ø^p < 0.05 compared to Western diet only, ^#^p<0.01 compared to Western Diet only, ^∞^p<0.001 compared to Western Diet only, and ‘p < 0.0001 compared to Western Diet only) among diets and B(a)P concentrations used.

In order to ascertain whether B(a)P alone and in combination with diet types will influence the production of inflammatory cytokines and markers of oxidative stress in their circulatory system, we analyzed potential increases of these proteins in B(a)P-treated rats that were on the WD versus the RD. Rats that received B(a)P in WD category had high cholesterol concentrations compared to RD at 25 and 50 μg/kg dose groups, while at 100 μg/kg the cholesterol concentrations were lower for WD compared to RD (Figure 7). When the data was compared across individual diet types, the 100 μg/kg B(a)P + RD had significantly higher concentrations of cholesterol than the 25 μg/kg group. On the other hand, the cholesterol concentrations were not significantly different in the WD group.

Plasma triglyceride levels showed a significant difference with the dietary type in controls and B(a)P treatment groups, with WD showing elevated levels compared to its RD counterparts. Rats that received WD + B(a)P registered increased levels of insulin in comparison to rats that received RD + B(a)P and also controls (Figure 7).

Levels of glucose stayed the same between WD and RD for 25-, and 50 μg B(a)P/kg bw, but showed a significant difference (p < 0.001) at 100 μg B(a)P/kg bw with WD group showing high concentrations (Figure 7). Levels of adiponectin did not vary between B(a)P + WD, and B(a)P + RD groups except at the 100 μg B(a)P dose (Figure 7). Leptin concentrations were shown to be increased among rats that were provided WD with the exception of groups dosed with 25 or 50 μg B(a)P/kg bw, which showed no significant difference between the dietary groups (Figure 7). On the other hand, there was no difference in C-reactive protein (CRP) levels between B(a)P + RD and B(a)P + WD for the 25 μg B(a)P/kg bw groups, but for 50 and 100 μg B(a)P/kg bw groups, the WD group registered low CRP levels compared to its RD counterparts (Figure 7).

Rats that received B(a)P and were on WD had low cholesterol concentrations compared to RD at 100 μg/kg bw. On the other hand, at 25 and 50 μg/kg B(a)P + WD dose groups, the cholesterol concentrations were greater than 25 and 50 μg/kg B(a)P + RD dose groups (Figure 7). Plasma triglyceride levels showed a significant difference with the dietary type in controls and B(a)P treatment groups, with WD showing elevated levels compared to its RD counterparts. Additionally, a dose-dependent relationship was observed with 100 μg/kg B(a)P + WD registering greater TG concentrations than the 25- and 50 μg/kg B(a)P + WD treatment groups (Figure 7).

Plasma glucose, insulin and leptin (Figure 7) levels showed significant increase in PIRC rats that received WD alone and 100 μg B(a)P/kg bw + WD. There was a lack of difference in tumor necrosis factor-alpha (TNF-α) and adiponectin concentrations, markers of pro- and anti-inflammatory functions respectively between B(a)P doses and diet groups (Figure 7). The levels of C-reactive protein (CRP), another proinflammatory molecule showed a significant decrease in PIRC rats that received WD compared to the RD (Figure 7).

## DISCUSSION

The objective of this study was to study how Western diet potentiates B(a)P-induced colon carcinogenesis in PIRC rats. The B(a)P doses employed in our study are relevant to human exposure scenarios as the levels of PAH are similar to levels that would be acquired in the diet. If a young adult weighing 50 kg were to eat a half-pound of fried chicken (that contains 5 μg/kg) or charcoal-broiled steak (that contains 9 μg/kg) every day, it translates to a dose of 30-50 ng/kg/day [16]. Some studies have reported a total PAH intake of 14 [17] and 59.2 μg/kg/day [18] and a recent report lists an intake of 371 μg/person/day [19]. As the B(a)P residue levels in dietary items show significant geographical variation (because of local contamination sources and inputs; [20]), the different analytical methodologies used, and the cooking practices adopted [21], the doses of 25, 50 and 100 μg B(a)P/kg body wt. administered to PIRC rats in the present study approximates to intake of B(a)P-contaminated foods for a period of 1-2 years. The above estimates hold true for fried meat eaters, who are non-smokers. A smoker will get an additional 3 μg B(a)P/pack of 20 cigarettes/day. For people who overindulge in fried/barbecued meat and smoke, the B(a)P doses administered to PIRC rats in our study are likely to fall within the range of human exposure to B(a)P.

The lack of effect of B(a)P on liver weights and liver enzymes observed in the present study is similar to that reported by our research group in rats [22] and other investigators in mice [23, 24] that were orally exposed to B(a)P. Benzo(a)pyrene treatment did not cause any damage to the liver cells as shown by the ALT & AST data. These liver enzymes are elevated in toxicant-induced injury settings as a result of leakage from cytosol across the damaged plasma membrane into the general circulation [25]. While ALT is specific for liver, AST is specific for extrahepatic tissues [26] and the ALT/AST ratio is considered to be indicative of hepatic/extra hepatic damage. The lack of variation in these enzyme activities between control and treatment groups suggest that B(a)P is not a liver toxicant, an observation that is supported by the histopathological evaluation of liver tissues that turned out to be normal (data not shown). Additionally, liver weight to body weight ratio did not show any appreciable difference between control and B(a)P treatment groups, indicating that liver hypertrophy did not occur as a result of B(a)P exposure and/or diet.

The metabolic and cellular changes that ensue following B(a)P exposure may create an environment that is conducive to inflammation, cell proliferation, and tumor growth in colon. While dietary toxicants like B(a)P have been reported to cause colon tumors [10, 27, 28], diet type ingested has been known to accelerate this process; diet rich in saturated fat was found to exacerbate the B(a)P-induced colon tumors [10, 29]. In vivo studies using a mouse model exposed to B(a)P indicated that diet enhanced the expression of inflammatory molecules such as IL-1β in liver and TNF-α in liver and colon [30]. In this context it must be noted that B(a)P has been implicated in several inflammation-related diseases including cancer and the interplay of several biological mechanisms act as drivers of the tumorigenic process [11].

Excess energy consumption through diet may have resulted in elevated levels of insulin, triglycerides and fatty acids, which subsequently induced colonic epithelial cells to proliferate and also expose them to reactive oxygen intermediates, a setting ideal for colon cancer progression [31]. Even though WD, because of its high calorific value, is expected to contribute to an increased amount of circulating cholesterol, compared to the RD, the concentrations did not differ in the control rats that were fed RD or WD. The lack of elevated levels of cholesterol in rats that were administered B(a)P and on WD is not unexpected because malignant cells and tumors were known to utilize higher than normal amounts of cholesterol [32, 33]. Since cancer cells require cholesterol in excess to maintain a high level of proliferation [34], the decline in serum cholesterol levels at high B(a)P dose group in PIRC rats could be attributed to the uptake of cholesterol by the growing colon tumors. Interestingly, rats in this dose group exhibited invasive neoplasia. Increased blood insulin and glucose are conducive to cancer cell growth. Insulin is known to increase IGF-1 levels, which decreases cancer cell death and increases cancer cell growth. Both insulin and sugars showed a pronounced effect in B(a)P high dose group, indicating their likely involvement in the progression and metastasis of colon cancer when B(a)P is taken in along with WD.

Diet-induced obesity was shown to have increased production of inflammatory cytokines and markers of oxidative stress in the circulatory system [35]. Even though the PIRC rats used in our studies did not appear to be obese, the greater visceral fat mass in WD rats compared to their RD counterparts could be due to accumulation of macrophages and a proinflammatory environment [36, 37]. The accumulated macrophages in adipose tissue may generate cytokines that signal the liver to produce inflammatory molecules as part of inflammatory response [38]. This response is reflected in a modest increase in circulating levels of inflammatory molecules, and insulin resistance that may have implications for colon tumor growth [39].

As adipose tissue is known to harbor both pro- and anti-inflammatory factors, we sought to measure the levels of leptin, and TNF as representatives of proinflammatory factors; CRP and adiponectin as representatives of anti-inflammatory factors. Leptin concentrations did not vary much between 25 and 50 μg/kg doses of B(a)P, but at 100 μg/kg dose the rats that were on WD showed a statistically significant increase compared to RD. Interestingly, leptin concentrations mirrored the trend similar to that of insulin, despite reports of leptin inhibiting insulin secretion in rodents [40, 41], which suggests the existence of other mechanisms for regulating leptin.

On the other hand, the levels of TNF-α, another proinflammatory molecule, failed to show any variation either with the diet type or B(a)P dose group. It has been reported that CRP exhibits pleiotropic effects and possesses both pro- and anti-inflammatory activities [42]. Case control studies by Gunter et al [43] showed that elevated CRP levels in blood may afford protection against early stages of tumorigenesis. Since our samples were collected at the end of B(a)P and diet exposure, by which time the adenomas were fully developed and the low CRP levels at the high B(a)P dose group signifies reduced systemic anti-inflammatory response and the immunosurveillance is compromised. Hence it could be surmised that the role of CRP is context-dependent and for our experimental regimen, the decrease in CRP levels indicates that it dampens the anti-inflammatory responses at high B(a)P doses for rats that were on WD.

The concentration of adiponectin, another anti-inflammatory cytokine that plays an important role in glucose and lipid metabolism and renders a protective effect against cancer [44], showed a trend similar to that of CRP. The lack of adiponectin effect at low doses of B(a)P suggest that adiponectin may render a protective effect at these doses to keep the colon cancer at bay by increasing the number of apoptotic cells, antiangiogenesis cytokines, and reduced the insulin resistance contributing to its anti-inflammatory and antitumor effects (Moon et al., 2013). At high B(a)P doses, adiponectin's deficiency contributes to inflammation-induced tumors through insulin resistance [46, 47]. Adiponectin also mediates induction of cyclogenase-2 (Cox-2), which is an early response gene involved in inflammatory diseases [48]. The uniform expression of Cox-2 paralleled with that of TNF-α, another inflammatory cytokine. The lack of variation in TNF-α indicate that TNF-α cannot modulate Cox-2 and Cox-2 is not a mechanism for WD-acceleration of B(a)P-induced CRC.

In terms of polyp phenotypes and pathology there were no major differences between the Apc*^Min^* mice used in our earlier studies [10] and the PIRC rat used in this study. The PIRC rat colon tumors were greater in number and larger in size compared to the Apc*^Min^* mouse. The rat model has a longer lifespan than the mouse model. The IHC results obtained from this study also imply that the same signaling pathways are activated in polyp formation in both these rodent models regardless of diet. They are a manifestation of the nature of the model (Apc mutation, increased Wnt activation) employed and are what one would expect to see in PIRC rats or Min mice (only a slight increase in TGF-β, for instance).

The variation in plasma adipokine levels could be attributed to the crosstalk of several adipokines in adipose tissue and other tissues (such as liver and intestines) contributing to metabolic dysregulation, which appears to be B(a)P-dose and diet type-dependent. Even though, the diet groups in conjunction with B(a)P showed a proinflammatory setting, the PIRC rats did not become obese. Our studies have implications for people, who are not obese but are at a high risk of metabolic dysfunction due to accumulation of visceral fat [49]. Studies have also shown that men with mild steatosis or non-alcoholic fatty liver disease registered greater levels of serum inflammatory markers than obese men without steatosis but with similar status of visceral adiposity [50], a situation that may also put these people at risk, should their dietary habits change with an increased intake of B(a)P as shown by our studies in PIRC rats. In this context, it should be mentioned that visceral fat mass (white fat) is known to sequester B(a)P and also induce CYP1B1, a CYP enzyme involved in B(a)P biotransformation [55]. As a result of this process, the delivery of B(a)P metabolites reaching colon in addition to the metabolites generated there [56] may accelerate colon tumor development.

To summarize, our studies have demonstrated that i) WD results in a greater number of colon tumors in Pirc rats compared to animals that were fed unsaturated fat/normal rodent chow; ii) WD results in accrual of visceral and subcutaneous fat depots, likely reservoirs for B(a)P accumulation; iii) WD does promote increased tumor formation in the rat when treated with B(a)P and iv) treatment with B(a)P and WD causes adenoma progression to high grade dysplasia in the PIRC rat colon. The tumor burden was associated with circulating cholesterol, triglyceride, glucose, insulin, leptin and adiponectin concentrations. The concordance between dietary fat, B(a)P dose, tumor load, invasive nature of adenomas, and the concentrations of the above-mentioned markers provide a compelling rationale that Western diet accelerates B(a)P-induced colon tumor formation and proliferation through enhanced insulin, leptin and other inflammatory molecules, which as a consequence may induce important signaling pathways such as PI3k/Akt and ERK1/2. As a next step, our studies will explore the underlying mechanisms of how WD modulates B(a)P biotransformation enzymes (CYP1A1, CYP1B1, glucuronosyltransferase, sulfotransferase, and glutathione-S-transferases) and increases the number of colon tumors compared to RD. Future studies will also include using flexible endoscopes to accurately measure the area/volume of colon tumors, assess the severity of colonic inflammation and monitor the tumor progression in PIRC rats on a temporal basis. These studies will help us understand if Western diet along with B[a]P contributes to an early onset of tumor development compared to regular diet with B[a]P.

## MATERIALS AND METHODS

### Animals, diets, B(a)P exposure

Seven-week-old PIRC rats were purchased from Taconic (Hudson, NY) or bred in house at the University of Missouri. Animals were cared for and housed in Accreditation of Laboratory Animal Care (AAALAC)-accredited animal care facility according to the recommendations established in the NIH Guide for the Care and Use of Laboratory Animals and the ARRIVE (Animal Research: Reporting *In Vivo* Experiments) guidelines [51]. The animals were maintained on a 12/12 hour light/dark cycle (lights on at 0600 hour) and allowed free access to either the AIN-76A diet (regular diet; RD; 5% fat content) or the Western diet (WD; 58% fat content) and water. The diets were purchased from the Research Diets, Inc. (Cat# D10001 and Cat# D12079B for RD and WD respectively). The PIRC rats were housed in groups of 2 per cage and allowed a seven-day acclimation period prior to being randomly assigned to the following treatment categories: I-AIN-76 diet (RD) only (n = 8), II-Western diet (WD) only (n = 8), III-B(a)P (25 μg/kg bw) + RD (n = 8), IV-B(a)P (50 μg/kg bw) + RD (n = 8), V-B(a)P (100 μg/kg bw) + RD (n = 8), VI-B(a)P (25μg/kg bw) + WD (n = 8), VII-B(a)P (50 μg/kg bw) + WD (n = 8), VIII-B(a)P (100μg/kg) + WD (n = 8). The number of animals used were determined after performing a “statistical power analyses” using PS software [52]. Power analysis revealed a minimum of 8 rats is required for each control or treatment group to detect a 20% change in experimental endpoints with 80% power and a type I error rate of 5%. Rats were administered B(a)P (97% pure, Sigma Chemical Co., St. Louis, MO Cat# B1760), dissolved in research grade tricaprylin (Sigma, Cat# T9126), daily through oral gavage at a volume of 300 μl for 60 days. As B(a)P is a potential carcinogen and mutagen, it was handled in accordance with NIH guidelines [53]. At the end of exposure period, rats were euthanized; and tissues of interest such as colon, liver, adipose, and other tissues were retrieved. Rats that were fed with the above mentioned-diets but received no B(a)P (categories I & II) served as controls.

### Measurement of body weight gain

To ensure that accurate B(a)P doses were being administered to rats in accordance to their body weight changes and also to examine if diet and/or B(a)P-treatment may have an effect on body weight gain or loss, we measured the weights of the rats over the 60 day period. Before dosing of rats, the weight of each rat was measured. Average weight of each previously described treatment groups were calculated at day 1 of the experiment and for every 7 days thereafter.

### Measurement of food consumption

In order to correlate weight gain with food consumption, we measured the amount of food consumed by the rats on a weekly basis. Before dosing of rats, the amount of food provided in each cage was measured. Each cage contained 200 grams of either AIN-76A diet (RD) or Western diet (WD). Average amount of food consumed was periodically estimated by measuring the amount of food retained in the feeding chamber before the food was replenished, the amount spilled and the amount given at the beginning of the feeding period for each cage.

### Extraction of blood samples for analysis of biochemical markers

At the end of the 60 day exposure, rats were anaesthetized via isoflurane (3%) and circulating blood was collected from the inferior vena cava using a butterfly needle and drained into a tube containing anticoagulant ethylenediaminetetraacetic acid (EDTA). The plasma was harvested from blood and analyzed for biochemical markers as detailed below.

The concentrations of plasma cholesterol and triglycerides were measured colorimetrically. Cholesterol kit (Cat # R80035) was obtained from Cliniqa (San Marcos, CA), and triglyceride kit (Cat #T7531-150) was obtained from Pointe Scientific (Canton, MI). The C-reactive protein (CRP) concentrations were measured using the rat CRP kit purchased from AssayPro (St. Charles, MO; Cat #ERC 1001-1). The adiponectin protein concentrations in serum were assayed using the rat adiponectin ELISA kit purchased from AssayPro (Cat #ERA 2500-1). The concentrations of insulin in serum were assayed using rat insulin RIA kit (Millipore, MA; Cat #RI-13K). The leptin concentrations were analyzed using the rat leptin ELISA kit (RayBiotech, GA; Cat #ELR-Leptin). The glucose concentrations were assayed using the kit procured from Cayman Chemical Co. (Ann Arbor, MI; Cat #10009582). TNF-alpha concentrations were assayed using the kit purchased from Ray Biotech (Norcross, GA; Cat #ELR-TNFa-1). In order to ascertain whether there is any tissue-specific damage associated with B(a)P exposure, the plasma samples were assayed for alanine aminotransferase (ALT) and aspartate aminotransferase (AST) using the kits purchased from Biomedical Research Service Center (University at Buffalo, NY; Cat# E-115 and Cat# E-116 respectively). All the biochemical marker assays were run in duplicate. Each sample, corresponding blank and standard (wherever appropriate) were analyzed using three replicates.

### Retrieval of tissues for adenoma scoring and histopathological analysis

After the 8 week-old Pirc rats were treated with B(a)P and diets for 60-days, colons and livers were harvested after euthanasia with exposure to isoflurane (6%). The colons were longitudinally opened and flushed of excreta using physiological saline. The size, location and number of adenomas were documented. The colons were Swiss rolled and preserved in 10% formalin for 24 hrs and then preserved in 70% ethanol until processing for observation of gross pathological changes. In addition to studying the total distribution of polyps in colon, we also categorized the polyps on the basis of their size. The neoplastic lesions were evaluated by size (<0.5 mm, >5.0 mm; using a digital Vernier caliper), number (single and multiple) and type (adenoma with or without high-grade dysplasia, or invasive adenocarcinoma). The processing of samples for routine histology (hematoxylin and eosin staining and sectioning) were done at the Translational Pathology Shared Resource of the Vanderbilt University Medical Center (TPSR-VUMC). Adenomas were characterized assessing for degree of dysplasia and classified as showing low or high grade dysplasia or adenocarcinoma. Determination of high grade dysplasia was based upon architectural complexity, higher nuclear/cytoplasm ratio, and loss of polarity as described by Boivin et al [54]. The pathologist who examined and assessed the slides was blinded to the treatment groups.

### Immunohistochemistry

Immunohistochemistry for β-catenin, caspase-3, anti-cyclin D1, anti-E-cadherin and Ki-67 was performed by at the TPSR-VUMC and for c-myc and Cox-2 by the Translational Pathology Core of the Vanderbilt GI SPORE.

All immunohistochemistry results were evaluated as follows. The intensity of the stain was graded as strong, moderate or weak, and the percentage of positive adenoma cells was scored semi-quantitatively as diffuse, ≥25%; focal, 1%-25%; negative, <1%.

For β-catenin, caspase-3, anti-cyclin D1, anti-E-cadherin and TGF β, the unstained slides were equilibrated to room temperature and then baked at 55°C for 1 hour. Slides were placed on the Bond Max IHC stainer (Leica Biosystems, Buffalo Grove, IL). All steps besides dehydration, clearing and coverslipping were performed on the Bond Max. Slides were deparaffinized and heat-induced antigen retrieval was performed on the Bond Max using their Epitope Retrieval 2 solution for 20 min. Slides were placed in a Protein Block (Dako North America, Inc., Carpinteria, CA; Cat # x0909,) for 10 min. The sections were incubated with either monoclonal or polyclonal antibodies to detect β-catenin (Cell Signaling Technology, Danvers, MA; Cat #9582) for one hour at a 1:100 dilution; caspase-3 (Cell Signaling Technology, Cat #9664,) for one hour at a 1:300 dilution; anti-cyclin D1 (ThermoFisher Scientific, Cat #RM-9104-S) for one hour at a 1:200 dilution; anti-E-cadherin (R&D Systems, Minneapolis, MN; Cat#AF748) for one hour at a 1:400 dilution and followed by a biotinylated anti-goat (Vector Laboratories, Inc., Burlingame, CA; Cat#BA-5000) for 30 minutes at a 1:200 dilution; TGF β Abcam, Cambridge, MA; (Cat#ab16667) diluted 1:100 for one hour. The Bond Refine Polymer detection system (Leica) was used for visualization. Slides were then dehydrated, cleared and coverslipped.

For Cox 2, and c-myc, the unstained slides were first de-paraffinized with xylene and then were hydrated through 100%, 90%, 70% and 50% ethanol. Antigen retrieval from the sections was performed by microwave treatment (104°C) for 20 min in citrate buffer (pH 6.0). The slides were allowed to cool at room temperature for 10 min in the citrate buffer. All slides were then quenched (to block the endogenous peroxidase) with 0.03% H_2_O_2_ with sodium azide for 5 min. Slides were blocked for 20 minutes with serum-free protein block (Dako), followed by application of primary antibody and were incubated for 60 min (cmyc) to overnight (for Cox 2) in a humidity chamber. The antibodies and dilutions used were: COX-2 Rabbit Monoclonal Antibody (Cell Signaling, Cat#12282) at a dilution of 1:600; c-myc Rabbit Monoclonal Antibody (Abcam, Cat#ab32072) at a dilution of 1:50; F4/80 Rabbit Monoclonal Antibody (Sigma, Cat#SAB5500103) at a dilution of 1:100). After incubation, the specimens were washed in phosphate-buffered saline (PBS) incubated for 30 min with the secondary biotinylated antibody followed by Dako EnVision + horseradish peroxidase labelled polymer (Dako) for another 30 min according to the manufacturer's instructions. Histological signal (a brown color) was developed using 3, 3-diaminobenzidine tetra hydrochloride (DAB, Dako kit Cat#k0411) in a chromogen solution for 5 minutes, washed in distilled water, and counterstained with haematoxylin for 1 min. All the procedures were performed at room temperature. For both markers, the known positive tissues and adenomas were used as positive controls. The primary antibody was omitted and replaced by phosphate buffered saline for negative controls. Sections were dehydrated and mounted using mounting medium (Dako) and then examined microscopically (Leica DM2500, Image Pro Plus 7.0 software) for positively stained cells.

### Statistical treatment of data

Data on polyp counts and biochemical markers were analyzed by two-way analysis of variance (ANOVA) to determine the effect of diet type and B(a)P dose. The differences among means were determined by using Bonferroni's post-hoc test. The criterion for statistical significance was set at *p*<0.05.
